# Data showing differential expression of Monocyte chemoattractant protein-1 in response to symptomatic and asymptomatic *T. vaginalis* infection

**DOI:** 10.1016/j.dib.2020.105628

**Published:** 2020-04-28

**Authors:** Sonal Yadav, Sumeeta Khurana, Rashmi Bagga, Rakesh Singh Dhanda, Manisha Yadav

**Affiliations:** a Dr. B. R. Ambedkar Center for Biomedical Research (ACBR), University of Delhi, Delhi 110007, India; b Department of Medical Parasitology, PGIMER, Chandigarh 160012, India; c Department of Obstetrics and Gynecology, PGIMER, Chandigarh 160012, India; d Stem cell laboratory, SMiLE incubator, Scheelevägen 2, 22381 Lund, Sweden; e Assistant Professor, Dr. B. R. Ambedkar Centre for Biomedical Research, University of Delhi, Delhi 110007, India.

**Keywords:** MCP-1/CCL-2, Symptomatic, Asymptomatic, *Trichomonas vaginalis*

## Abstract

Trichomoniasis is caused by *Trichomonas vaginalis* (a protozoan parasite). About 80% of the infected cases remain asymptomatic [1]. The differential response of showing symptoms or no symptoms is not yet explored. However, some studies gave us some insights on the pathogenesis of trichomonas and also about host defense mechanism. Host secretes pro-inflammatory cytokines and chemokines to evade infection. Monocyte chemoattractant protein-1 (MCP-1/CCL2) is a strong chemoattractant of monocytes, NK-cells and T-lymphocytes. Many reports have shown high MCP-1 levels during trichomonas infection [2], [3], [4], [5] in human prostate stromal myofibroblast cells (WPMY-1), HeLa cells, vaginal epithelial cells (VECs) but levels in response to symptomatic and asymptomatic isolates is not yet reported. In this article, we have reported MCP-1 levels in the vaginal washes and serum samples of BALB/c mouse infected with symptomatic and asymptomatic *T. vaginalis* isolates for different time points. We found higher levels of MCP-1 in vaginal washes of symptomatic group on 2nd day post infection (dpi) than control uninfected group. While on 4th dpi and 14th dpi, higher levels of MCP-1 in vaginal washes was observed in asymptomatic group as compared to control group. However, significant level of MCP-1 was observed in asymptomatic group on 14th dpi as compared to symptomatic group in vaginal washes. We have also observed significantly higher levels of MCP-1 in the serum samples of symptomatic group on 2nd, 4th and 14th dpi as compared to control group. A higher level of MCP-1 was found at all the time points in serum samples of asymptomatic group as compared to control group. Interestingly, a significant higher level of MCP-1 was found in the serum samples of BALB/c mice in asymptomatic as compared to symptomatic group. The MCP-1 levels in both vaginal washes and serum were significantly higher in asymptomatic group at later time points.

Specifications table

**Table:** 

Subject	Biological Sciences
Specific subject area	Medical Microbiology (Parasitology), *Trichomonas vaginalis*
Type of data	Graphs
How data were acquired	MCP-1 levels in symptomatic and asymptomatic *T. vaginalis* infected group by ELISA assay.
Data format	RAW, Analyzed
Parameters for data collection	BALB/c female mouse (6–8 weeks), *T. vaginalis* isolates obtained from symptomatic and asymptomatic patients.
Description of data collection	Serum and vaginal washes were collected from symptomatic and asymptomatic *T. vaginalis* infected and control uninfected BALB/c mice.
Data source location	University of Delhi, New Delhi, India
	Postgraduate Institute of Medical Education and Research (PGIMER), Chandigarh, India
Data accessibility	Data is with this article only.

## Value of the data

• First comparative report showing MCP-1 levels in response to symptomatic and asymptomatic T. vaginalis infection.
• Significant level of MCP-1 was observed at later time points in asymptomatic infected group as compared to symptomatic group.
• Increased levels of MCP-1 were observed in symptomatic group at early time points, which gradually decreased later on.
• Information about pro-inflammatory cytokine profiling in response to symptomatic and asymptomatic T. vaginalis can be helpful in understanding the role of cytokines and chemokines in generating symptoms or no symptoms in these subjects.

## 1 Data description

**Fig. 1:**
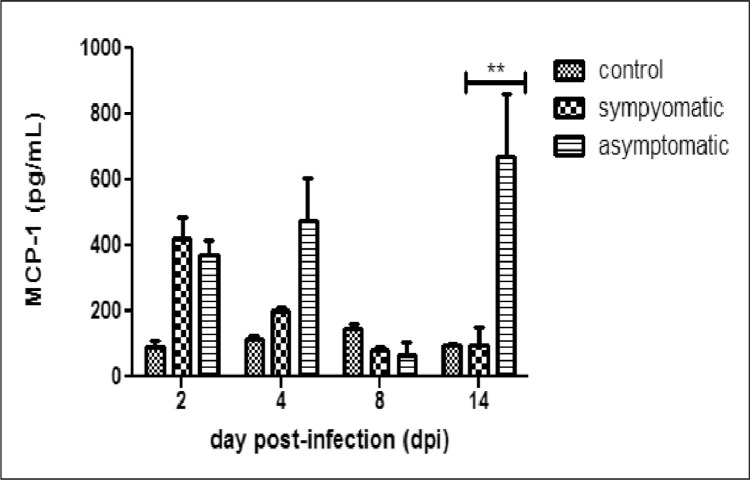
MCP-1 levels in the vaginal washes of BALB/c mice infected with symptomatic and asymptomatic *T. vaginalis* isolates and control uninfected group at different time points (**p < 0.01).

**Fig. 2:**
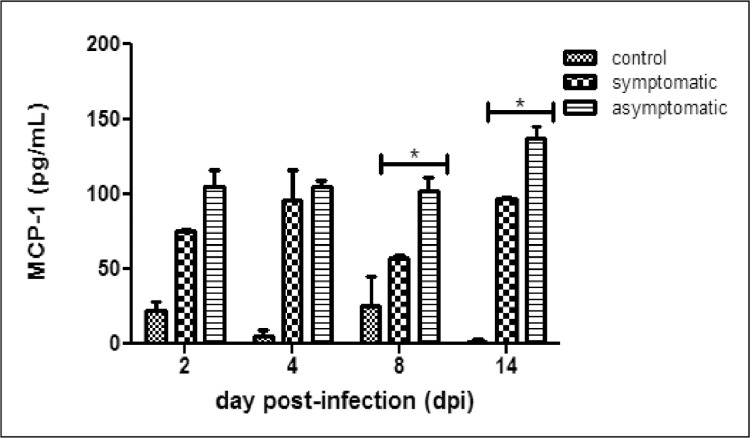
MCP-1 levels in the serum samples of BALB/c mice infected with symptomatic and asymptomatic *T. vaginalis* isolates and control uninfected group at different time points (*p < 0.05).

Assay of MCP-1 levels in serum and vaginal washes of BALB/c mice infected with symptomatic and asymptomatic isolates of *T. vaginalis* and in control uninfected was observed. Higher level of MCP-1 was observed in the vaginal samples of BALB/c mice infected with symptomatic isolates as compared to control group. MCP-1 level in the symptomatic group was 418 pg/mL, in asymptomatic group was 368 pg/mL and in control uninfected group was 90 pg/mL on 2nd dpi (Fig. 1). In symptomatic group gradual decreased levels of MCP-1 was observed on later time points. Significantly higher level of MCP-1 was found in asymptomatic group (673 pg/mL) as compared to symptomatic group (97 pg/mL) on 14th dpi (*P* < 0.01) (Fig. 1). Significantly higher level of MCP-1 was observed in the serum samples of BALB/c mice infected with asymptomatic isolates as compared to symptomatic on 8th dpi (102 pg/mL verses 57 pg/mL) and 14th dpi (137 pg/mL verses 97 pg/mL) respectively (*P* < 0.05) (Fig. 2).

## 2 Experimental design, materials, and methods

### 2.1 T. vaginalis strains

*T. vaginalis* symptomatic and asymptomatic strains were cultured in the Department of Medical Parasitology, PGIMER, Chandigarh, India. Trophozoites were subcultured in Diamond's TYI-S-33 medium (pH 6.8) supplemented with 10% heat-inactivated horse serum and 1% penstrep at 37 °C for 3 days before inoculation in the mouse model.

### 2.2 L. acidophilus strain

L. *acidophilus* MTCC 10,307 was purchased from the Institute of Microbial Technology (IMTECH), Chandigarh and cultured in Man, Rogosa Sharp (MRS) broth (Himedia) in 5% CO_2_ incubator at 37 °C at 5000 rpm for overnight.

### 2.3 Measurement of MCP-1 levels

All the experiments were performed according to the animal ethical guidelines and prior permission was taken from Institutional Animal Ethics Committee (Ref. No. 82/IAEC/525). For the measurement of MCP-1 levels, six-eight week old, female BALB/c mice were obtained from the animal facility of PGIMER, Chandigarh and inoculated with 50 µl of 10^8^ trichomonads per ml intra-vaginally in mice for two consecutive days after Estradiol and L. *acidophilius* exposure [6]. Animals were divided into three groups: Control group (no infection), symptomatic and asymptomatic infected group. Further, vaginal washes and serum samples were collected after the infection on 2nd dpi, 4th dpi, 8th dpi and 14th dpi and MCP-1 level was measured by ELISA (BidScientific, E-EL-M0006).

## 3 Statistical analysis

The MCP-1 concentration was interpolated by using Graphpad Prism statistical software. Duplicate sets of each sample were included; data of all the samples is shown as mean ± standard deviation. Two way analysis of variance (ANOVA) followed by bonferroni assay was used for statistical comparison between the three groups. Confidence interval (CI) is considered as 95% of confidence. *P* values ≤0.05 were considered as statistically significant.

## Appendix Supplementary materials

Image, application 1

Image, application 2
