# Supplementary material for: Data showing differential expression of Monocyte chemoattractant protein-1 in response to symptomatic and asymptomatic T. vaginalis infection
Source: Data Brief. 2020 Apr 28;30:105628. doi: 10.1016/j.dib.2020.105628 (PMC7210420; doi:10.1016/j.dib.2020.105628)
Supplement: Supplementary file 2 [file mmc2.pdf]

Standard Curve

**Conc.**	**OD values**	
pg/ml	OD 1	OD 2
1000	3.545099974	3.573400021
500	2.846999884	3.010999918
250	2.361500025	2.299299955
125	1.461500049	1.695500016
62.5	1.024600029	0.957000017
31.25	0.667599976	0.611899972
15.63	0.3653	0.369700015
0	0.070600003	0.071300001

**Sym**	symtomatic
**Asym**	asymptomatic

OD values of the serum samples

	duplicate value of sample 1		duplicate value of sample 2	
Sym 2dpi	1.158599973	1.104099989	1.185700059	1.328199983
Sym 4dpi	2.029299974	1.299499989	1.024399996	1.168900013
Sym 8dpi	0.947300017	0.906899989	0.843500018	0.827300012
Sym 14dpi	1.672199965	1.16170001	1.696699977	1.150099993
Asym 2dpi	1.631700039	1.701300025	1.074800014	0.978699982
Asym 4dpi	1.54489994	1.611700058	1.330199957	1.154099941
Asym 8dpi	1.232599974	1.511000037	1.691699982	1.452900052
Asym 14dpi	1.875	1.814200044	2.278100014	1.708699942
Control 2dpi	0.533	0.51160001	0.352800001	0.372800003
Control 4dpi	0.149693333	0.147706664	0.29890001	0.260900018
Control 8dpi	0.766900011	0.726900033	0.24249	0.202509993
Control 14dpi	0.193600007	0.201600007	0.119500002	0.095500002

OD values of the serum samples

	sample 1		sample 2	
	average OD	average OD-blank	average OD	average OD-blank
Sym 2dpi	1.131349981	1.060399981	1.144900024	1.073950024
Sym 4dpi	1.664399981	1.593449981	1.161949992	1.090999992
Sym 8dpi	0.927100003	0.856150003	0.875200003	0.804250003
Sym 14dpi	1.416949987	1.345999987	1.429199994	1.358249994
Asym 2dpi	1.666500032	1.595550032	1.38805002	1.31710002
Asym 4dpi	1.578299999	1.507349999	1.470950007	1.400000007
Asym 8dpi	1.371800005	1.300850005	1.601350009	1.530400009
Asym 14dpi	1.844600022	1.773650022	2.046150029	1.975200029
Control 2dpi	0.522300005	0.451350005	0.362800002	0.291850002
Control 4dpi	0.148699999	0.077749999	0.279900014	0.208950014
Control 8dpi	0.746900022	0.675950022	0.222499996	0.151549996
Control 14dpi	0.197600007	0.126650007	0.107500002	0.036550002

MCP-1 levels in serum samples

	sample 1	sample 2	Mean (1&2)	STDEV	SEM
Sym 2dpi	74.87692	75.91924	75.39807711	0.737025182	0.521155504
Sym 4dpi	115.8808	77.23077	96.55576822	27.32967651	19.32499959
Sym 8dpi	59.16539	55.17308	57.16923101	2.822987808	1.996153822
Sym 14dpi	96.84615	97.78846	97.31730696	0.666312496	0.471154085
Asym 2dpi	116.0423	94.62308	105.3326943	15.14568399	10.70961585
Asym 4dpi	109.2577	101	105.1288464	5.839069782	4.128845838
Asym 8dpi	93.37308	111.0308	102.2019236	12.48587419	8.828846308

Asym 14dpi	129.7423	145.2462	137.4942327	10.96287512	7.751923341
Control 2dpi	28.02692	15.75769	21.89230796	8.675656432	6.134615495
Control 4dpi	0	9.380771	4.690385169	6.633206319	4.690385169
Control 8dpi	45.30385	4.965384	25.13461609	28.52360109	20.16923175
Control 14dpi	3.05	0	1.525000271	2.156676066	1.525000271

OD values of the vaginal washes samples

	duplicate value of sample 1		duplicate value of sample 2	
Sym 2dpi	0.247500002	0.280800015	0.206499994	0.220899999
Sym 4dpi	0.190400004	0.121100001	0.174099997	0.123499997
Sym 8dpi	0.088799998	0.106200002	0.103	0.1043
Sym 14dpi	0.085000001	0.081200004	0.131400004	0.131300002
Asym 2dpi	0.162699997	0.239800006	0.233500004	0.241400003
Asym 4dpi	0.210199997	0.209999993	0.298999995	0.32069999
Asym 8dpi	0.145400003	0.075400002	0.075800002	0.073299997
Asym 14dpi	0.391000003	0.405000001	0.215700001	0.31310001
Control 2dpi	0.121100004	0.105100004	0.096599999	0.0926
Control 4dpi	0.110999846	0.131200123	0.130399998	0.090399998
Control 8dpi	0.144899988	0.124899988	0.123050001	0.117950014
Control 14dpi	0.117600001	0.097600013	0.115000031	0.098399975

OD values of the vaginal washes samples

	sample 1		sample 2	
	average OD	average OD-blank	average OD	average OD-blank
Sym 2dpi	0.264150009	0.193200009	0.213699996	0.142749996
Sym 4dpi	0.155750003	0.084800003	0.148799997	0.077849997
Sym 8dpi	0.0975	0.02655	0.10365	0.0327
Sym 14dpi	0.083100002	0.012150002	0.131350003	0.060400003
Asym 2dpi	0.201250002	0.130300002	0.237450004	0.166500004
Asym 4dpi	0.210099995	0.139149995	0.309849992	0.238899992
Asym 8dpi	0.110400002	0.039450002	0.074549999	0.003599999
Asym 14dpi	0.398000002	0.327050002	0.264400005	0.193450005
Control 2dpi	0.113100004	0.042150004	0.094599999	0.023649999
Control 4dpi	0.121099985	0.050149985	0.110399998	0.039449998
Control 8dpi	0.134899988	0.063949988	0.120500007	0.049550007
Control 14dpi	0.107600007	0.036650007	0.106700003	0.035750003

MCP-1 levels in vaginal washes samples

	sample 1	sample 2	Mean (1&2)	STDEV	SEM
Sym 2dpi	484.85	351.9	418.375	94.00984656	66.475
Sym 4dpi	210.45	193	201.725	12.33901333	8.725
Sym 8dpi	75.95	88.85	82.4	9.121677477	6.45
Sym 14dpi	44.85	151.05	97.95	75.09474016	53.1
Asym 2dpi	323.35	413.55	368.45	63.78103166	45.1
Asym 4dpi	342.95	608	475.475	187.4186524	132.525
Asym 8dpi	106.1	27.4	66.75	55.64930368	39.35
Asym 14dpi	859.05	487.2	673.125	262.9376566	185.925
Control 2dpi	110	70.3	90.15	28.07213921	19.85
Control 4dpi	128.1	103.3	115.7	17.53624817	12.4
Control 8dpi	160.3	128.1	144.2	22.76883835	16.1
Control 14dpi	98.85	96.6	97.725	1.590990258	1.125
